# Diffusion imaging changes in grey matter in Alzheimer’s disease: a potential marker of early neurodegeneration

**DOI:** 10.1186/s13195-015-0132-3

**Published:** 2015-07-01

**Authors:** Philip S.J. Weston, Ivor J.A. Simpson, Natalie S. Ryan, Sebastien Ourselin, Nick C. Fox

**Affiliations:** Dementia Research Centre, Institute of Neurology, The National Hospital for Neurology and Neurosurgery, Box 16, Queen Square, London, WC1N 3BG UK; Centre for Medical Image Computing, The Engineering Front Building, University College London, Malet Place, London, WC1E 6BT UK

## Abstract

Alzheimer’s disease (AD) is recognized to have a long presymptomatic period, during which there is progressive accumulation of molecular pathology, followed by inexorable neuronal damage. The ability to identify presymptomatic individuals with evidence of neurodegenerative change, to stage their disease, and to track progressive changes will be important for early diagnosis and for prevention trials. Despite recent advances, particularly in magnetic resonance imaging, our ability to identify early neurodegenerative changes reliably is limited. The development of diffusion-weighted magnetic resonance imaging, which is sensitive to microstructural changes not visible with conventional volumetric techniques, has led to a number of diffusion imaging studies in AD; these have largely focused on white matter changes. However, in AD cerebral grey matter is affected very early, with pathological studies suggesting that grey matter changes predate those in white matter. In this article we review the growing number of studies that assess grey matter diffusivity changes in AD. Although use of the technique is still at a relatively early stage, results so far have been promising. Initial studies identified changes in diffusion measures in the hippocampi of patients with mild cognitive impairment, which predated macroscopic volume loss, with positive predictive value for progression to AD dementia. More recent studies have identified abnormalities in multiple neocortical areas (particularly the posterior cingulate) at various stages of disease progression. Studies of patients who carry genetic mutations predisposing to autosomal dominant familial AD have shown cortical and subcortical grey matter diffusivity changes several years before the expected onset of the first clinical symptoms. The technique is not without potential methodological difficulties, especially relating to partial volume effects, although recent advances appear to be reducing such issues. Going forward, further utilization of grey matter diffusion measurements in AD may improve our understanding with regards to the timing and nature of the earliest presymptomatic neurodegenerative changes. This imaging technique may also be useful in comparing and contrasting subtle variations in different disease subgroups, and as a sensitive outcome measure for presymptomatic clinical trials in AD and other neurodegenerative diseases.

## Introduction

There is growing evidence in Alzheimer’s disease (AD) of a long latency period, with pathological changes beginning decades before symptom onset [1, 2]. During this period there is progressive accumulation of molecular pathology, followed by increasing and inexorable neuronal damage. A ‘self-perpetuating’ aspect of neurodegeneration that is difficult to slow once established may account, at least in part, for notable clinical trial failures [3, 4]. There is increasing recognition that our best chance of maintaining brain function may be to offer therapies as early as possible, when the minimum of irretrievable neuronal loss has occurred, and when there is potential to prevent or delay the onset of cognitive decline [5]. In order to do this, methods are needed to identify individuals at risk, to stage their disease, and to track progression with sensitive and robust measures [6].

Significant advances in AD biomarkers and neuroimaging measures, in particular, have been made over the past two decades. These measures have helped improve both our understanding of the disease and our ability to detect and monitor pathological changes in research and clinical settings. The earliest magnetic resonance imaging (MRI) studies focused on macroscopic brain changes, with the best established symptomatic and late presymptomatic marker being hippocampal atrophy [7–9]. However, subsequent studies have shown that the predictive value of such measures, particularly when applied as a single cross-sectional measure at the level of the individual, is limited prior to the onset of dementia [10, 11]. More recently, automated techniques to measure cortical thickness have shown early changes in multiple cerebral areas [12–14]. However, the utility of these techniques in the earliest presymptomatic stages is again uncertain. There currently appears to be a gap of several years between the appearance of the earliest pathological changes (for example, of amyloid deposition using positron emission tomography or cerebrospinal fluid (CSF) measures) and the point when conventional macroscopic imaging techniques are first able to detect degenerative change reliably.

**Fig. 1:**
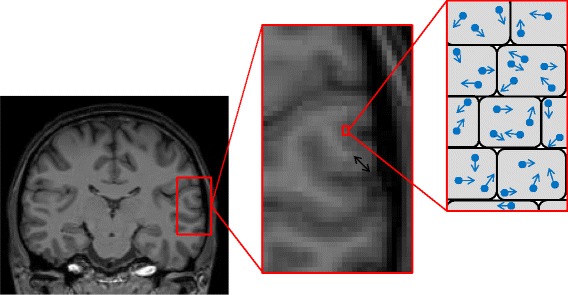
Coronal view of a T1-weighted magnetic resonance image of the brain (left image); magnified area of cortex, with the black arrow indicating the cortical thickness (center); on the right is a schematic representation of a magnified region of cortex, with water molecules diffusing within the cells, dependent on the integrity of the cell structure

In recent years, there have been great advances in diffusion-weighted imaging [15]. The key benefit of diffusion MRI is the ability to observe changes at the microscopic level (Fig. 1). The breakdown of microstructural barriers, such as myelin, cell membranes and intracellular organelles, which would normally restrict the Brownian motion of water molecules, results in a measureable difference in the diffusion of water molecules [16]. Such changes are not visible on conventional structural MRI sequences, and several studies suggest they predate macroscopic atrophy [17–19].

**Fig. 2:**
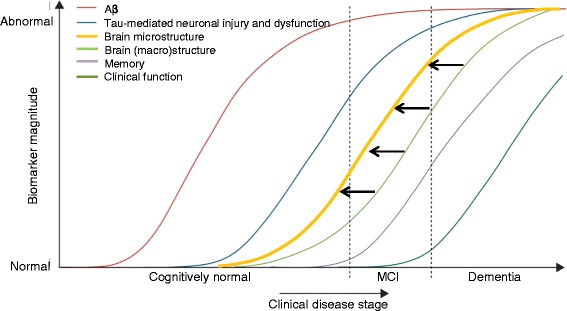
A graphical illustration of the sequence of biomarker changes that are thought to occur in Alzheimer's disease prior to the clinical manifestation of dementia (adapted from the model proposed by Jack *et al*. [55]). An additional curve has been added (in orange) to represent where microstructural brain changes (the earliest of which are likely to occur in the grey matter) are predicted to lie in the overall sequence. The black arrows show how the use of magnetic resonance imaging to detect diffusivity changes in grey matter may allow significantly earlier detection of neurodegenerative change than is possible with conventional (macro)structural imaging methods. Aβ, amyloid β; MCI, mild cognitive impairment

The initial focus of diffusion-weighted MRI in neurodegeneration research was on the study of white matter tract integrity, using the diffusion tensor model [16]. This was commonly assessed using the metric of fractional anisotropy, which describes the directional coherence of diffusion along fibers. However, whilst studying the breakdown of white matter structural connectivity has helped broaden our understanding of AD, the specific mechanisms underlying white matter damage remain unclear [19–21]. One suggestion has been that white matter changes are the result of Wallerian degeneration, a downstream consequence following the earlier loss of cortical neurons [20]. Also, from a histopathological standpoint, AD is primarily a cortical disease, particularly in the early stages [22]. Grey matter changes have also been shown to correlate more closely with clinical abnormalities than white matter changes [23], and have a closer link to clinical symptoms than amyloid deposition [2, 24]. The application of diffusion imaging for the detection of microscopic grey matter abnormalities may therefore be a potentially powerful tool in identifying the earliest AD changes (Fig. 2).

A growing body of literature is emerging that describes the use of diffusion-weighted MRI to detect microscopic grey matter changes in a number of neurodegenerative disorders. Indeed in one such condition - Creutzfeldt-Jakob disease (CJD) - detection of diffusivity changes in grey matter has now become the gold standard in clinical diagnostic practice [25, 26]. Alzheimer’s disease is a less rapidly progressive condition than CJD, and so diffusivity changes might not be as marked. However, the use of diffusion MRI for the detection of grey matter abnormalities has shown promise in less rapidly progressive conditions, such as multiple sclerosis, where it was used to demonstrate the existence of cortical grey matter damage [27].

More recently diffusion-weighted imaging of grey matter in AD has been successfully used in a number of different studies. The majority of studies have used the metric of mean diffusivity (MD), which assesses the average degree of diffusion in all directions, as opposed to quantifying directionality. It has been argued that, compared with fractional anisotropy, MD lends itself better to the assessment of cortical and subcortical grey matter, where net diffusion may not be expected to conform to any one specific direction [28]. As cellular microstructure breaks down and there are fewer obstacles to diffusion, molecules are able to diffuse more freely and MD is generally expected to increase [15]. We will now review the results of these studies and consider both what they tell us about the pathophysiology of AD, and how they might be utilized in the clinical setting.

## Method of systematic literature review

A literature search was conducted on PubMed in October 2014. We aimed to identify all articles that used diffusion-weighted MRI to investigate grey matter diffusivity changes in AD. The search [Alzheimer’s] AND [DTI OR diffusion] AND [grey matter OR cortex] was applied. The title and abstract of all articles identified by the search were assessed for suitability for inclusion in the review. For all suitable articles from the initial search, the titles and abstracts of articles in their reference lists were reviewed, and any additional suitable articles also included.

## Hippocampal diffusivity

Early studies of grey matter diffusion assessed MD in the hippocampi of patients with amnestic mild cognitive impairment (MCI), and compared the imaging results of those who did and did not go on to progress to clinical AD [17, 18]. In these studies, which contained 18 (Muller *et al*. [18]) and 24 (Kantarci *et al*. [17]) amnestic MCI patients, hippocampi were segmented manually, with care taken to ensure that none of the surrounding CSF was included. The studies found that those who did progress to AD had significantly higher hippocampal MD at baseline than those who did not. Also, compared with macroscopic volume measurements, MD was a more sensitive predictor of progression to clinical 'AD dementia', and also correlated significantly better with severity of episodic memory deficits. These findings demonstrated that microscopic changes are detectable within the hippocampi prior to definite volumetric change, thus offering potential improvement in diagnostic sensitivity. Muller and colleagues [29–31] went on to replicate these findings and showed that the manual segmentation and analysis technique had good intra-observer and inter-observer reliability.

Douaud *et al*. [32] used an automated voxel-wise analysis to assess both grey and white matter diffusion, again in a cohort of amnestic MCI patients. They also found hippocampal diffusivity to be a sensitive predictor of progression over a 3 year follow-up period, with it being more sensitive than any changes to white matter tracts, and also a better predictor than CSF A-beta amyloid and tau measurements.

## Beyond the hippocampi - neocortical changes in diffusivity

Following the finding of early hippocampal diffusivity changes, the focus has broadened to other cortical areas. Rose *et al*. [33] used whole brain voxel-wise analysis to assess cortical changes in clinically established AD. A number of cortical areas, beyond the hippocampi, were found to have elevated MD. Regions affected included the posterior cingulate cortex (with the greatest effect size), entorhinal cortex, amygdala, parahippocampal gyrus, middle temporal gyrus, superior and middle frontal gyrus and the supramarginal gyrus bilaterally; a pattern which is similar to that seen in studies of cortical thickness [12]. A further study used both whole brain and manually segmented region of interest analyses to assess patients with MCI and with established AD [34]. A significant trend was seen along the trajectory from normal controls, to MCI, to established AD, in terms of whole brain average grey matter MD. This was contrary to volume measurements, which were not able to accurately predict progression. Additionally, MD measurements in a number of regions of interest, including hippocampi, amygdala, parieto-occipital association cortices and frontal lobe cortical areas, were found to be independently associated with disease progression. As discussed by the study’s authors, the observed spreading of microstructural cortical involvement as the disease progresses fits well with the established histopathological staging of AD [22].

Diffusion analysis of multiple cortical areas has demonstrated some value in differentiation of different types of dementia [35]. Compared with patients with dementia with Lewy bodies, patients with probable AD had higher MD in the hippocampi, parahippocampal gyri, tempero-parietal association cortices, posterior cingulate cortex and the precuneus. In a logistic regression model the ability to differentiate the two diseases was significantly increased with the addition of MD measurements compared with volume measurements alone.

Patients with earlier stages of AD have also shown widespread patterns of change. One study [36] of 20 patients with amnestic MCI found elevated diffusivity (with associated reduced fractional anisotropy) compared with controls, in a cortical distribution very similar to that described above. Again, the most marked effects were seen in the posterior cingulate cortex. Unfortunately, the value of cortical diffusivity measurements, and particularly that of the posterior cingulate, in predicting conversion from MCI to AD was not assessed. However, the importance of the posterior cingulate cortex in maintaining function in multiple different cognitive domains has been confirmed by a large cortical diffusion study of healthy individuals and patients with different focal MCI syndromes [37]. The study found that diffusivity changes in the posterior cingulate were independently associated with memory, language, executive function and visuospatial function.

## Familial Alzheimer’s - findings from presymptomatic disease

Although rare, autosomal dominant familial AD (FAD) shares many aspects, both molecularly and clinically, with sporadic AD [5]. Studying individuals from families affected by FAD provides the opportunity for prospective longitudinal study of individuals who are cognitively normal but who are known to be destined to develop the disease, thereby allowing assessment of preclinical neurodegenerative changes [5]. Whilst studies using diffusion MRI in preclinical FAD have involved relatively small samples, the findings have been informative.

A region of interest study of presenilin 1 (*PSEN1*) gene mutation carriers (n = 10) found significant differences in hippocampal diffusivity compared with controls [38]. In the symptomatic phase, hippocampal MD was elevated, as has been seen in sporadic AD. In the presymptomatic phase (5.6 years, on average, prior to predicted age at onset) hippocampal MD was also found to be abnormal and occurred prior to change in hippocampal volume.

In addition to the hippocampal changes described above, in a small study of *PSEN1* mutation-positive individuals, widespread neocortical diffusion changes were demonstrated prior to the onset of symptoms [39]. These changes were most prominent posteriorly, affecting the precuneus, posterior cingulate cortex and inferior parietal cortex, similar to that which has been seen in early symptomatic sporadic patients [36]. This distribution differed from symptomatic FAD patients, in whom MD changes were observed throughout the whole cortex.

The two studies of FAD described above also showed diffusivity changes in thalamus and caudate several years prior to predicted symptom onset, albeit with some changes in macroscopic volume measurements also being evident [38, 39]. The presence of early changes in subcortical grey matter integrity is consistent with the early subcortical amyloid deposition observed in familial AD, and serves to emphasize that it is not only cortical grey matter that is affected early in the disease process [40, 41]. However, whether assessment of similar subcortical grey matter areas would be useful in demonstrating early changes in sporadic AD, or whether early thalamostriatal change is unique to familial AD, has not yet been established.

**Fig. 3:**
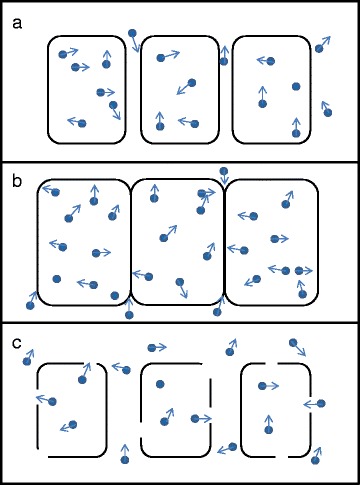
A simplified schematic representation of molecular diffusion in and around neurons, and how this may change over time in Alzheimer’s disease (AD). **a** In the early presymptomatic stage water molecules are able to diffuse normally, with the mean diffusivity (MD) being the same as a normal healthy individual. **b** Evidence from familial AD studies suggests that in the period shortly before symptom onset the MD falls, implying that diffusion is restricted. This restriction may be a result of cellular hypertrophy and/or inflammation, in response to amyloid deposition in the presymptomatic phase. **c** During the symptomatic phase, progressive cellular atrophy results in a breakdown in the usual barriers to diffusion, with studies showing an increase in MD compared with normal controls. The effects in (c) are likely to progressively outweigh the effects in (b) as the disease progresses

The above studies detected *in vivo* presymptomatic microstructural grey matter changes in limbic cortex, neocortex and subcortical grey matter structures. However, one key finding, consistent across all of these anatomical structures, was that the presymptomatic change in MD was not in the direction one would have expected, in that it was decreased rather than increased compared with controls. This is opposite to the direction of change observed in symptomatic AD (both familial and sporadic). This reduction in presymptomatic MD was also associated with marginally increased cortical thickness. Although unexpected, and requiring further replication in larger studies, the finding of a presymptomatic fall in MD is very interesting. It may suggest the presence of more than one pathological process affecting diffusion imaging changes: the presymptomatic reduction in MD may indicate an inflammatory response to amyloid accumulation (Fig. 3), occurring prior to (or coincident with and obscuring) the onset of microstructural breakdown and macrostructural atrophy [38, 39]. A summary of research studies assessing grey matter diffusivity in AD is given in Table 1.

**Table 1:** A summary of AD studies that have measured grey matter diffusion changes

Study	Familial or sporadic	n	Methods	Main findings
Kantarci *et al*. 2005 [17]	Sporadic	21 MCI, 54 NC	Hippocampi manually segmented. Volume and MD measured. 36 month clinical follow-up	Hippocampal MD better than hippocampal volume for predicting conversion from MCI to AD over the 36 month follow-up
Muller *et al*. 2005 [18]	Sporadic	18 MCI, 18 NC	Hippocampi manually segmented. Volume, MD + FA measured	Increased MD in hippocampus is strongest independent predictor of episodic memory decline, and is more sensitive than volume loss
Fellgiebel *et al*. 2006 [31]	Sporadic	18 MCI	Hippocampi manually segmented. Volume and MD measured. 18 month clinical follow-up with convertors and non-convertors compared	Increased left hippocampal MD at baseline in convertors compared with non-convertors
Rose *et al*. 2008 [33]	Sporadic	13 AD, 13 NC	Voxelwise GM MD analysis	Elevated MD in hippocampus, amygdala, and medial temporal, parietal, and frontal GM in AD. Largest number of abnormal voxels in PCC
Scola *et al*. 2010 [34]	Sporadic	21 AD, 21 MCI, 20 NC	Whole brain GM + WM MD; followed by ROI analysis. 2 year clinical follow-up with MCI convertors and non-convertors compared	Trend seen over normal/MCI/AD for GM + WM MD. Volume alone could not predict convertors, but diffusivity could
Kantarci *et al*. 2010 [35]	Sporadic	30 AD, 30 DLB, 60 NC	ROI analysis using FLAIR diffusion imaging, measuring MD (plus volumes) in GM	Compared to DLB, AD has elevated MD in hippocampi, parahippocampal gyri, amygdala, temporoparietal association cortices, PCC + precuneus. Measuring MD increases ability to identify AD
Douaud *et al*. 2013 [32]	Sporadic	35 MCI	Voxelwise GM MD measured, with convertors and non-convertors compared (36 month follow-up)	Elevated left hippocampal and amygdala MD in convertors. Left hippocampal MD was the best predictor of conversion
Jacobs *et al*. 2013 [36]	Sporadic	20 MCI, 20 NC	Whole-brain CTh, MD analysis on GM. ROI analysis then applied	MCI showed increased MD in precuneus, PCC, supramarginal + frontal cortices; largest effect in the PCC
Fortea *et al*. 2010 [39]	Familial	6 PMC, 5 SMC, 18 NC	ROI analysis of cortical and subcortical GM MD (plus CTh and subcortical volumes)	Reduced MD (plus CTh) in precuneus, PCC + parietotempral association cortices in PMCs Widespread elevated MD in SMCs
Ryan *et al*. 2013 [38]	Familial	10 PMC, 10 SMC, 20 NC	ROI MD and FA (GM and WM) + GM volumes	In PMC, reduced MD in right hippocampus (without atrophy) + cingulum, with increased FA in thalamus and caudate. In SMCs MD rises

AD, Alzheimer’s disease; CTh, cortical thickness; DLB, Dementia with Lewy bodies; FA, fractional anisotropy; GM, grey matter; MCI, mild cognitive impairment; MD, mean diffusivity; NC normal control; PCC, posterior cingulate cortex; PMC, presymptomatic mutation carrier; ROI, region of interest; SMC, symptomatic mutation carrier; WM, white matter

## Methodological considerations

Measuring molecular diffusion in grey matter structures is not without pitfalls. An important issue is the potential for results to be biased by partial volume effects [18, 35]. This problem is particularly important in cortical studies because the cortex has a thickness (generally 2 to 3 mm) that is similar to the typical voxel diameters used in diffusion imaging (2 to 2.5 mm isotropic), with the issue being complicated further by the fact that the cortex has a convoluted structure and is adjacent to CSF spaces. There is, therefore, a danger of cortical voxels also containing some CSF, with this risk increasing as the cortex atrophies.

Investigators have addressed the issue of potential partial voluming in a number of ways. Accurate segmentation, ensuring the region does not include any CSF, is important [29, 31, 33]. Setting inclusion thresholds that exclude any voxel with an MD approaching that of CSF has also been done [33]. However, altering the characteristics of the distribution of MD measures by thresholding based on those same measures is questionable; this can give distinctly non-Gaussian distributions, which are difficult to analyze statistically. A further method used to reduce the risk of partial voluming has been to co-register the diffusion images to a FLAIR sequence, as opposed to a T2 sequence as is conventionally done [35, 37]. By doing this, the signal from the CSF is suppressed, increasing the reliability of cortical diffusivity measurements. An additional consideration relates to how the diffusion image is spatially co-registered to the segmentations of the structural scan, which is itself not a trivial process, and can introduce further partial voluming if not done accurately [42, 43].

A methodological development that offers potential benefits for the measurement of grey matter diffusivity is the recent advent of multiband MRI [44]. By simultaneously acquiring magnetic resonance data from multiple slices, this technique significantly increases the acquisition speed and offers the possibility of reducing the voxel size - from the 2 to 2.5 mm isotropic that is currently most common down to 1 to 1.5 mm isotropic - without having to substantially increase the duration of the scan. This improvement in spatial resolution significantly reduces partial voluming, and is increasingly used [45, 46]. New multi-shell acquisition techniques, including composite hindered and restricted model of diffusion (CHARMED) and neurite orientation dispersion and density imaging (NODDI), are able to model neural tissue in terms of multiple separate compartments (for example, intracellular, extracellular and CSF) [47, 48]. This allows these techniques to model the partial volume effect, which is not possible with conventional single-shell techniques, and therefore offers potential for more precise and reliable diffusion metrics.

An additional factor to consider if grey matter diffusion is to enter widespread clinical use is the significant time and effort required to segment regions of interest manually. Grey matter diffusivity analysis would ideally be automated [49]. Such methodology has recently been used successfully in studies of cortical and subcortical grey matter diffusivity in AD and other neurodegenerative conditions [38, 50, 51], and could potentially be transferred to the clinical setting.

## Future directions

Whilst detection of grey matter diffusivity changes in AD does show promise as a potential early biomarker, the number and size of the studies performed to date is relatively limited. Therefore, further replication of results, ideally in large multicentre cohorts, will be very important.

One interesting and potentially useful direction of investigation would be to develop a cortical 'signature' of microstructural change for early AD, with the aim of identifying a characteristic pattern of specific cortical regions that undergo the earliest changes. This approach has already been employed successfully for macrostructural measurements in AD [12].

Diffusion imaging may prove powerful in detecting differences in underlying disease mechanisms, with one study having already demonstrated significant differences between different underlying neurodegenerative pathologies [35]. Further studies of this type, where AD is compared with other disease processes rather than with healthy controls only, are likely to improve our ability to differentiate between different diseases in the clinical setting.

A further area of study that may improve sensitivity to the earliest neurodegenerative changes in AD would be the measurement of grey matter diffusivity across serial MRI scans in order to assess longitudinal change. Measurement of within-individual changes avoids between-individual variability and may be a more sensitive and relevant marker of pathological change than a single assessment [52]. Tracking of within-individual longitudinal change in diffusivity may also be useful when it comes to presymptomatic, or early symptomatic, therapeutic trials. Whilst it is unlikely that such trials will rely on any one biomarker alone, with assessment of a combination of different imaging, CSF and neuropsychometric measures likely to be the optimal approach [53, 54], the potential inclusion of grey matter diffusivity measurements could prove valuable. The use of grey matter diffusivity measurement in combination with other molecular and neurodegeneration markers will also help improve our understanding of how the timing of microstructural breakdown fits in with other pathological changes.

The finding that MD initially decreases in the presymptomatic stage of FAD [38, 39], prior to increasing as microstructural breakdown occurs, warrants further investigation in larger presymptomatic imaging studies, as well as additional *in vitro* investigation to replicate and ascertain the likely underlying mechanism.

## Conclusion

Diffusion imaging provides a means of assessing *in vivo* microstructural changes in the brain; such changes are likely to predate the macrostructural atrophy that characterizes neurodegenerative disorders such as AD. The most striking example of this is in CJD where diffusion imaging of grey matter has proven to be a remarkably sensitive and specific marker of disease. Diffusion changes in grey matter in other, less rapidly progressive disorders have been relatively understudied, but are increasingly of interest in AD given that the earliest pathological changes appear in grey matter in this disease and the growing acceptance of a long prodromal period when molecular pathology accumulates and yet cerebral atrophy can be difficult to detect.

Grey matter diffusivity has shown initial promise in the detection of early presymptomatic changes, as well as in prediction of conversion from MCI to AD, and in the differentiation of different dementia subtypes. Grey matter structures commonly implicated, including the hippocampus and posterior cingulate, have been identified as parts of a network vulnerable to early AD pathological changes. Measuring diffusion changes in the grey matter 'nodes' of these networks may provide information that is complementary to the more studied changes in white matter. However, whilst initial results appear positive, current literature is relatively limited, with findings to date focusing on cross-sectional group changes only. It is therefore true that a note of caution is required when looking to the future. That said, magnetic resonance diffusion acquisition capabilities and analytical techniques are advancing rapidly. These advances offer the prospect of grey matter diffusivity adding to our understanding of the evolution of early changes in AD and other neurodegenerative disorders - and contributing to clinical diagnosis or providing inclusion or outcome measures for trials.

## Abbreviations

AD: Alzheimer’s disease
CJD: Creutzfeldt-Jakob disease
CSF: cerebrospinal fluid
FAD: familial Alzheimer’s disease
MCI: mild cognitive impairment
MD: mean diffusivity
MRI: magnetic resonance imaging
